# Luteolin Enhances Transepithelial Sodium Transport in the Lung Alveolar Model: Integrating Network Pharmacology and Mechanism Study

**DOI:** 10.3390/ijms241210122

**Published:** 2023-06-14

**Authors:** Lei Chen, Tong Yu, Yiman Zhai, Hongguang Nie, Xin Li, Yan Ding

**Affiliations:** 1 Department of Pharmacology, School of Pharmacy, China Medical University, Shenyang 110122, China; lchen@cmu.edu.cn; 2 Department of Stem Cells and Regenerative Medicine, College of Basic Medical Science, China Medical University, Shenyang 110122, China; 3 Department of Chemistry, School of Forensic Medicine, China Medical University, Shenyang 110122, China; 4 Liaoning Province Key Laboratory of Forensic Bio-Evidence Sciences, Shenyang 110122, China; 5 Center of Forensic Investigation, China Medical University, Shenyang 110122, China

**Keywords:** phytochemicals, network pharmacology, acute lung injury, 3D alveolar epithelial organoid, epithelial sodium channel, JAK/STAT pathway

## Abstract

Luteolin (Lut), a natural flavonoid compound existing in *Perilla frutescens* (L.) Britton, has been proven to play a protective role in the following biological aspects: inflammatory, viral, oxidant, and tumor-related. Lut can alleviate acute lung injury (ALI), manifested mainly by preventing the accumulation of inflammation-rich edematous fluid, while the protective actions of Lut on transepithelial ion transport in ALI were seldom researched. We found that Lut could improve the lung appearance/pathological structure in lipopolysaccharide (LPS)-induced mouse ALI models and reduce the wet/dry weight ratio, bronchoalveolar protein, and inflammatory cytokines. Meanwhile, Lut upregulated the expression level of the epithelial sodium channel (ENaC) in both the primary alveolar epithelial type 2 (AT2) cells and three-dimensional (3D) alveolar epithelial organoid model that recapitulated essential structural and functional aspects of the lung. Finally, by analyzing the 84 interaction genes between Lut and ALI/acute respiratory distress syndrome using GO and KEGG enrichment of network pharmacology, we found that the JAK/STAT signaling pathway might be involved in the network. Experimental data by knocking down STAT3 proved that Lut could reduce the phosphorylation of JAK/STAT and enhance the level of SOCS3, which abrogated the inhibition of ENaC expression induced by LPS accordingly. The evidence supported that Lut could attenuate inflammation-related ALI by enhancing transepithelial sodium transport, at least partially, via the JAK/STAT pathway, which may offer a promising therapeutic strategy for edematous lung diseases.

## 1. Introduction

In acute lung injury (ALI), the injury to the cells of alveolar epithelium may lead to an inflammatory storm and progressive diseases. Pathological specimens from patients with ALI or its severe form—acute respiratory distress syndrome (ARDS)—often show diffuse alveolus damage, with an accumulation of inflammatory edema fluid; thus, the effective clearance of superabundant fluid is necessary for restoring the gas exchange in the alveoli [1,2]. Alveolar epithelial cells are composed mainly of alveolar epithelial type (AT) 1 and 2 cells, the latter of which are involved in the effect of secretion and regeneration to maintain lung homeostasis [3,4]. Bearing the potential of self-renewal and differentiation into AT1 cells, AT2 cells can maintain alveolar function and are identified to be the facultative progenitor cells in lung tissue repair during ALI [5]. The epithelial sodium channel (ENaC) consists mainly of α/β/γ subunits and is responsible for transporting Na^+^ from the apical to the basolateral side, thus regulating the water reabsorption of alveolar epithelial cells [6].

Lipopolysaccharide (LPS), a major biologically active component of the Gram-negative bacterial cell wall, can induce the features of acute inflammation in lung epithelium and facilitate extensive tissue damage in the organs, such as kidney, heart, and liver [7,8,9]. In LPS-induced ALI, ENaC expressed in AT2 cells can regulate the transepithelial sodium transport, ensuring the fluid clearance in edematous alveoli [2,10]. However, despite several important advances in ALI treatment during the last few decades, the specific mechanisms of ENaC-involved regulation are still undetermined. Therefore, discovering new drugs and therapeutic targets remains an urgent priority.

Organoids are three-dimensional (3D) structures derived from stem or progenitor cells, the extracellular matrix of which possesses the fundamental composition and function of multiple organs [11]. The aforementioned attributes render them a promising candidate for both fundamental research and clinical diagnosis/treatment. At present, the lung alveolar organoid has been a new pathological model for studying cell communication and host–pathogen interactions and is a powerful platform for simulating lung diseases, which can replace some animal experiments and, thus, minimize the use of animals in respiratory research [12].

Luteolin (Lut) (3′, 4′, 5, 7-tetrahydroxyflavonoids), a bioactive polyphenolic compound, can be extracted from many medicinal plants and some common vegetables/fruits, including *Perilla frutescens* (L.) Britton [13]. According to the Chinese medical system, *P. frutescens* can be used ethnically to treat respiratory problems, such as cold, fever, nasal congestion, and cough [14]. Fifteen types of compounds, including Lut, apigenin, rosmarinic acid, and caffeic acid, were separated and extracted from the *P. frutescens* leaves [15]. Among them, Lut is a flavonoid compound, which possesses numerous beneficial pharmacological actions, including anti-inflammatory, anti-oxidant, anti-viral, anti-tumor, and other biological properties [16,17,18]. Previous studies on ALI caused by sepsis or cecal ligation puncture have shown that Lut has protective therapeutic effects [19,20]. The mechanisms of Lut treatment in ALI have been uncovered to be related to inhibit the inflammatory reaction, including reducing the pulmonary reactive oxygen sepsis, whereas the actions of Lut on transepithelial ion transport in pneumonedema were seldom researched [21].

Network pharmacology is an innovation in elucidating the intricate progress of pathophysiology by evaluating the interactions among herbs, components, targets genes, and diseases. It is helpful in understanding the instinctive laws of formulas and revealing various targets for traditional Chinese medicine actions [22]. In this research, we confirmed the actions of Lut on the expression level of ENaC in primary AT2 cells, as well as constructed a molecular docking model to study the potential mechanism in ALI treatment. Of note, we established a lung alveolar model using a 3D alveolar epithelial organoid that was similar to the actual in vivo state in order to explore a new strategy of Lut treatment in edematous lung diseases.

## 2. Results

### 2.1. Lut Increased the Expression Level of ENaC in Primary AT2 Cells

**Figure 1:**
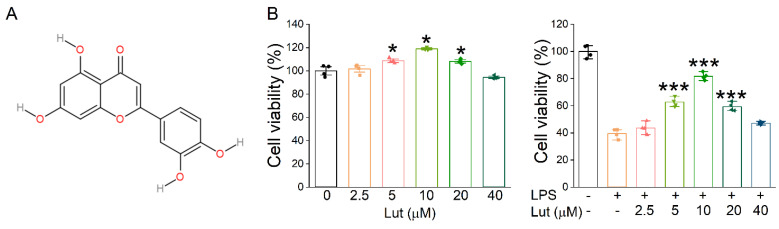
Effects of Lut on cell proliferation inhibited by LPS. (**A**) The molecular structure of Lut. (**B**) Left panel: Cell viability was detected after incubation with Lut (0, 2.5, 5, 10, 20, and 40 μM). * *p* < 0.05, compared with 0 μM group, *n* = 4. Mann–Whitney U test was used to analyze the difference of the means for significance. Right panel: Lut induced LPS-inhibited primary AT2 cell proliferation. *** *p* < 0.001, compared with LPS group, *n* = 4. One-way ANOVA followed by Bonferroni’s test was used to analyze the difference of the means for significance.

The molecular structure of Lut is shown in Figure 1A. Firstly, we evaluated the effect of Lut on primary AT2 cell viability by CCK8 assay, and the results showed that 5, 10, and 20 μM Lut could promote the proliferation of AT2 cells treated without or with LPS (Figure 1B, *p* < 0.05 vs. 0 μM group, *p* < 0.001 vs. LPS group). Notably, 10 μM of Lut showed the highest protective effect, which was used as the optimal treatment concentration for the subsequent cell experiments. However, with the further increases in concentration, 20 and 40 μM seemed to show lesser cell viability upon treatment with LPS, possibly due to the cytotoxicity, which may counteract the visibly proliferative effects.

**Figure 2:**
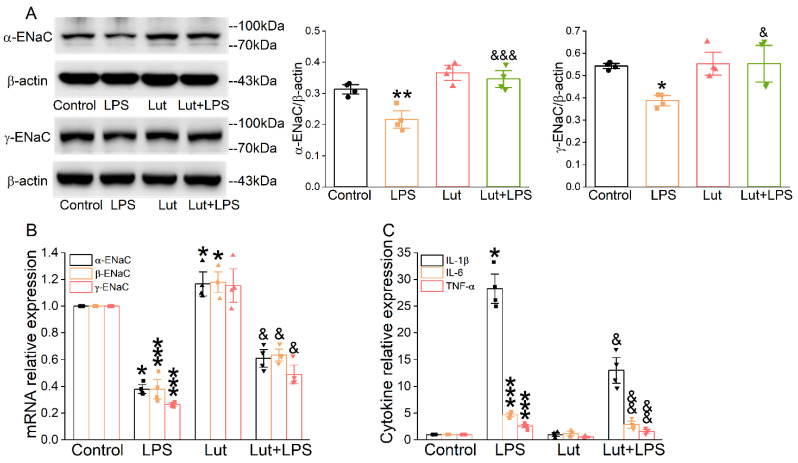
Lut increased the expression levels of ENaC and inhibited inflammation in primary AT2 cells. Cells were treated with Lut (10 μM, 24 h), co-presence or absence of LPS (10 μg/mL, 12 h). (**A**) Representative Western blot and corresponding graphical representation of data obtained from Western blot assays for α/γ-ENaC in primary AT2 cells, where bands were quantified using gray analysis (α, γ-ENaC/β-actin). * *p* < 0.05, ** *p* < 0.01, compared with Control group. ^&^ *p* < 0.05, ^&&&^ *p* < 0.001, compared with LPS group, *n* = 4. One-way ANOVA followed by Bonferroni’s test was used to analyze the difference of the α-ENaC means for significance. Mann–Whitney U test was used to analyze the difference of the γ-ENaC means for significance. (**B**) The expression levels of α/β/γ-ENaC mRNA were examined by real-time PCR with GAPDH set as the internal standard. * *p* < 0.05, *** *p* < 0.001, compared with Control group. ^&^ *p* < 0.05, compared with LPS group, *n* = 4. Mann–Whitney U test was used to analyze the difference of the α/γ-ENaC means for significance. One-way ANOVA followed by Bonferroni’s test was used to analyze the difference of the β-ENaC means for significance. (**C**) IL-1β, IL-6, and TNF-α levels in primary AT2 cells. * *p* < 0.05, *** *p* < 0.001, compared with Control group. ^&^ *p* < 0.05, ^&&^ *p* < 0.01, compared with LPS group, *n* = 4–5. Mann–Whitney U test was used to analyze the difference of the IL-1β means for significance. One-way ANOVA followed by Bonferroni’s test was used to analyze the difference of the IL-6/TNF-α means for significance.

As shown in Figure 2A, LPS reduced the quantity of α/γ-ENaC protein in AT2 cells compared with the Control group (*p* < 0.01~0.05), which was prominently alleviated after the Lut administration (*p* < 0.001~0.05 vs. LPS group). β-ENaC expression was not checked due to the lack of a suitable commercial antibody. Real-time polymerase chain reaction (PCR) assay showed that Lut eliminated the decrease in α/β/γ-ENaC mRNA induced by LPS (Figure 2B, *p* < 0.05 vs. LPS group), indicating that Lut may attenuate ALI by upregulating the expression of ENaC to improve transepithelial sodium transport. Consistently, the real-time PCR assay showed that LPS significantly increased the expression levels of inflammatory cytokines (Figure 2C, *p* < 0.001~0.05 vs. Control group), which were inhibited by Lut (*p* < 0.01~0.05 vs. LPS group).

### 2.2. Lut Suppressed Inflammatory Pulmonary Edema in ALI Mice

Through the prediction website pkCSM [23], we determined that the pharmacokinetic parameters for the logarithmic ratio of the partition coefficient (LogP), volume of distribution (VD_ss_ Log L/kg), total clearance (CL_tot_ Log mL/min/kg), and oral acute toxicity (LD_50_ mol/kg) were 2.2824, 1.153, 0.495, and 2.455, respectively. The results showed that Lut is a small molecule with good pharmacokinetic properties, exhibiting high intestinal absorption (>80%) and extensive distribution. Additionally, it serves as a substrate of P-glycoprotein and exhibits poor distribution in the brain. The in silico evaluation indicated that Lut has the potential to be developed as a pharmacological agent with suitable intestinal absorption and low toxicity.

**Figure 3:**
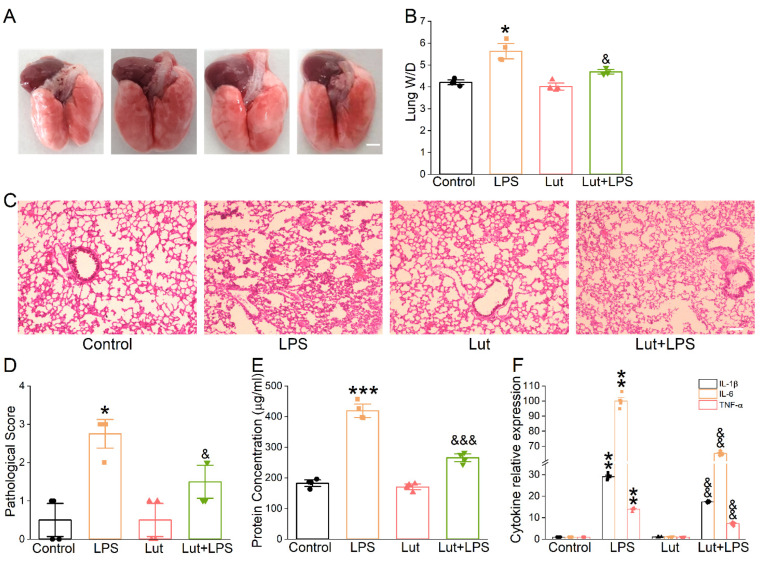
Effect of Lut on morphological structure and inflammation in LPS-induced ALI. Lut (20 mg/kg) was administered intraperitoneally to BALB/c mice 12 h before and after LPS (5 mg/kg) stimulation. (**A**) Representative photographs of whole lungs from all the experimental groups. Scale bar = 0.5 cm. (**B**) The lung W/D ratio was calculated as wet weight/dry weight. * *p* < 0.05, compared with Control group; ^&^ *p* < 0.05, compared with LPS group, *n* = 4. Mann–Whitney U test was used to analyze the difference of the means for significance. (**C**) The effect of Lut was assessed by H&E staining. Scale bar = 200 μm. (**D**) Quantifying lung injury scores in the lungs. * *p* < 0.05, compared with Control group. ^&^ *p* < 0.05, compared with LPS group, *n* = 4. Mann–Whitney U test was used to analyze the difference of the means for significance. (**E**) Protein content in BALF of mouse lung. *** *p* < 0.001, compared with Control group. ^&&&^ *p* < 0.001, compared with LPS group *n* = 4. One-way ANOVA followed by Bonferroni’s test was used to analyze the difference of the means for significance. (**F**) IL-1β, IL-6, and TNF-α levels in BALF of LPS-induced ALI. ** *p* < 0.01, compared with Control group. ^&&^ *p* < 0.01, compared with LPS group, *n* = 6. Mann–Whitney U test was used to analyze the difference of the means for significance.

To confirm the possible therapeutic action of Lut on ALI mice caused by LPS, we observed the appearance of the lungs and performed H&E for morphological research to evaluate the changes in histology. As expected, the photographs of LPS-treated lungs showed punctate hemorrhage and decreased surface smoothness, which were improved after the Lut administration (Figure 3A). Moreover, Lut significantly eliminated the LPS-induced increase in the lung wet/dry weight (W/D) ratio (Figure 3B, *p* < 0.05 vs. LPS group), which further implied that Lut could relieve the degree of pulmonary edema in ALI mice. Lung tissues in LPS group were significantly damaged, which were characterized by hemorrhage, inflammatory cell infiltration, and increased alveolar wall thickness and which were evidenced by an increased lung injury score (Figure 3C,D, *p* < 0.05 vs. Control group). As expected, Lut alleviated the histopathology changes in LPS-induced ALI mice (*p* < 0.05 vs. LPS group).

Alteration of the alveolar–capillary barrier was evaluated by bronchoalveolar lavage fluid (BALF) protein concentration, which was reduced significantly by Lut (Figure 3E, *p* < 0.001 vs. LPS group). Meanwhile, Lut could reverse the LPS-increased inflammatory cytokines (Figure 3F, *p* < 0.01 vs. LPS group), supporting that inflammation reaction existed in the LPS-induced ALI model and that one of the beneficial effects of Lut may be associated with inflammation-related edema formation.

### 2.3. Establishment of the 3D Alveolar Epithelial Organoids

**Figure 4:**
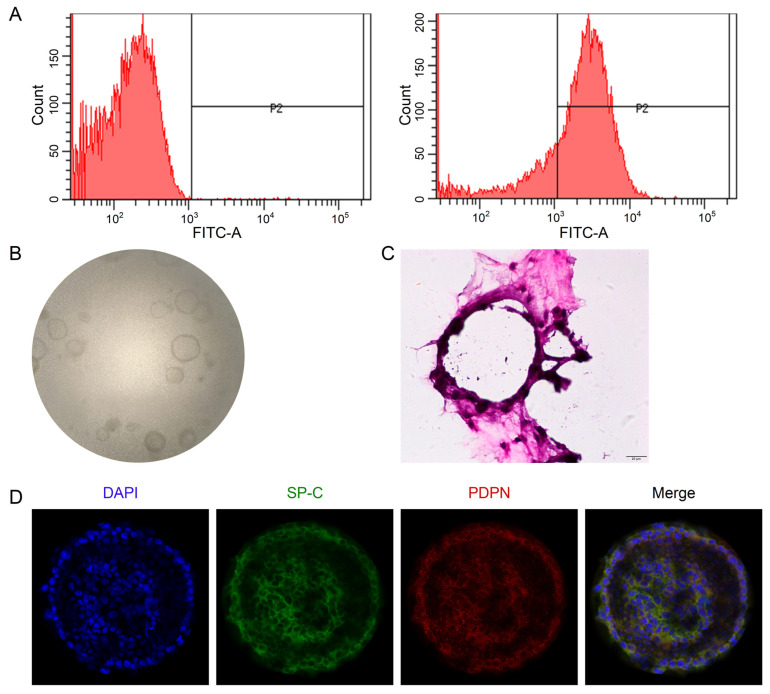
Establishment of 3D alveolar epithelial organoid model. (**A**) Representative data of flow cytometry for the purity of primary mouse AT2 cells (81.33 ± 5.02%), *n* = 3. (**B**) Representative DIC images of 3D organoid cultured for 8 days (40×). (**C**) H&E staining of 3D organoid culture. Scale bar, 20 μm. (**D**) Representative images of confocal images for 3D organoid stained with AT1 (PDPN)/AT2 (SP-C) markers. Scale bar, 50 μm.

The flow cytometry data showed that the purity of primary mouse AT2 cells was 81.33 ± 5.02% (Figure 4A), available for future co-culture with mouse lung fibroblasts. The 3D organoid cultures could be visualized between Days 4 and 12, and the number/size gradually increased over the culture time (Figure 4B). To better identify the 3D structure of alveolar epithelial organoids, we stained them with H&E and AT1 (PDPN)/AT2 (SP-C) markers, respectively. As shown in Figure 4C,D, monolayer-like alveolar epithelial cells were formed, and the confocal tomography identified that both AT1 and AT2 cell markers were expressed, suggesting that the lung alveolar model was successfully established.

### 2.4. Lut Elevated the Expression of ENaC in the Lung Alveolar Model

**Figure 5:**
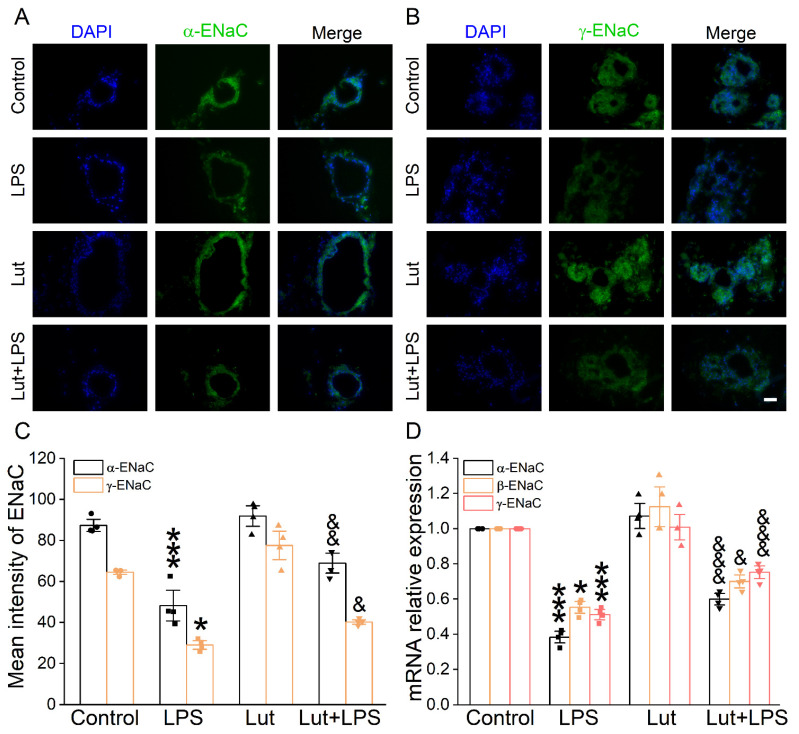
Effect of Lut on ENaC expression in 3D organoid culture. (**A**,**B**) Immunofluorescence staining showed the effect of LPS and Lut on the expression of α/γ-ENaC in 3D organoid culture. Scale bar, 50 μm. (**C**) Statistical diagram of α/γ-ENaC protein expression. * *p* < 0.05, *** *p* < 0.001, compared with Control group. ^&^ *p* < 0.05, ^&&^ *p* < 0.01, compared with LPS group, *n* = 4. One-way ANOVA followed by Bonferroni’s test was used to analyze the difference of the α-ENaC means for significance. Mann–Whitney U test was used to analyze the difference of the γ-ENaC means for significance. (**D**) The expression levels of α/β/γ-ENaC mRNA were examined by real-time PCR in 3D organoid culture. * *p* < 0.05, *** *p* < 0.001, compared with Control group. ^&^ *p* < 0.05, ^&&&^ *p* < 0.001, compared with LPS group, *n* = 4. One-way ANOVA followed by Bonferroni’s test was used to analyze the difference of the α/γ-ENaC means for significance. Mann–Whitney U test was used to analyze the difference of the β-ENaC means for significance.

To verify the influence of Lut on the transepithelial sodium transport close to the in vivo ALI state, we used 3D alveolar epithelial organoid immunofluorescence assay to detect the ENaC protein expression level. The green fluorescence intensity of α/γ-ENaC in the LPS group was significantly lower than that in Control group (Figure 5A–C, *p* < 0.001~0.05), which was enhanced after the Lut administration (*p* < 0.01~0.05 vs. LPS group), identifying that Lut could strengthen the salt water absorption in LPS-induced ALI. Moreover, Lut reversed the LPS-reduced α/β/γ-ENaC mRNA expression in the alveolar model (Figure 5D, *p* < 0.001~0.05 vs. LPS group).

### 2.5. Target Identification and Protein–Protein Interaction Analysis

**Figure 6:**
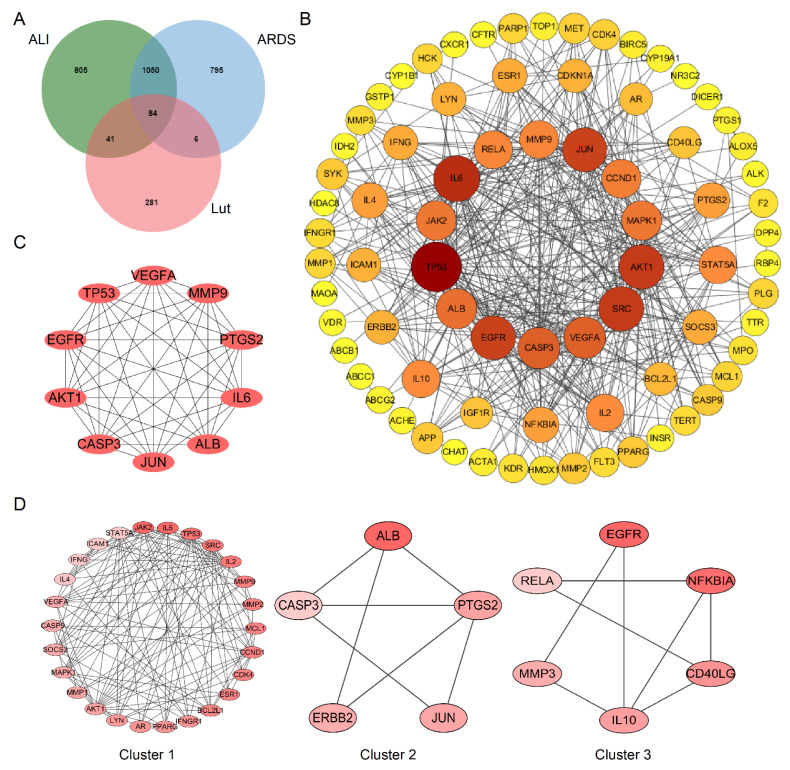
Bioinformatics analysis of overlapping genes. (**A**) Venn diagram of 84 overlapping genes between the predicted targets of Lut and the targets associated with ALI/ARDS. (**B**) PPI network of the 84 overlapping genes. The importance of each gene was analyzed by STRING. The higher the degree, the more crucial the gene. The degree of the outermost circle is 0–10, the middle circle is 11–20, and the smallest circle is >20. The colors of nodes from reddish to yellowish are arranged in descending order on the basis of their degree values. (**C**) Cytohubba, the plug-in of Cytoscape, was used to analyze the top 10 hub gene network of target proteins by MCC algorithm. (**D**) Cluster of the 84 overlapping gene-containing PPI network. Three clusters were identified. Cluster 1 had the highest score of 10.08, and JAK2 was identified as the seed gene. Cluster 2 had a score of 3.5, and ALB was the seed gene. Cluster 3 had a score of 3.2, and EGFR was the seed gene.

A total of 412 putative gene targets and 1980/1935 disease gene targets related to Lut and ALI/ARDS, respectively, were obtained, and the duplicates were removed. The protein targets were transformed to gene targets by the UniProt database. The intersection of putative Lut and ALI/ARDS disease targets resulted in 84 overlapping genes (Figure 6A), which were related to the Lut involvement of ALI/ARDS.

The protein–protein interaction (PPI) network was established using the String database to construct a drug–disease target interaction network. After removing free proteins that did not interact, there were 77 nodes (targets) and 430 edges (interactions) in the PPI network. The node size and color were positively correlated with degree, suggesting that darker and larger nodes played an important effect in the fight against ALI/ARDS (Figure 6B).

### 2.6. Screening of the Hub Genes and Clusters in the PPI Network

We used the MCC algorithm based on Cytoscape plug-in Cytohubba to screen out the top 10 Hub genes of target proteins and constructed the map of Hub gene network. TP53, VEGFA, MMP9, PTGS3, IL6, ALB, JUN, CASP3, AKT1, and EGFR were predicted to be the important targets for Lut intervention of ALI/ARDS (Figure 6C). We obtained three clusters using MCODE, among which Cluster 1 had the highest score of 10.080, and JAK2 was the seed gene (Figure 6D).

### 2.7. Prediction of the Potential Signaling Pathways of Lut for ALI Treatment

**Figure 7:**
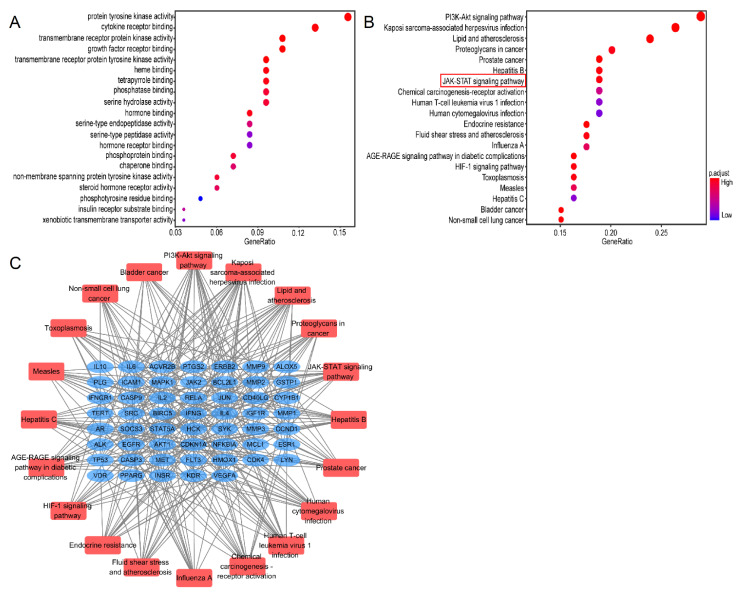
GO and KEGG analysis of 80 common genes. (**A**) A bubble chart of top 20 enriched GO items of potential targets in the molecular function. (**B**) Bubble chart of top 20 KEGG signaling pathways related to the effect of Lut against ALI. The redder the bubble, the smaller the P-value; the larger the bubble, the greater the number of genes that participated in this pathway. (**C**) The critical signaling pathway target visualization network. PPI network of the top 20 KEGG signaling pathways and associated target genes. Red nodes represent top 20 KEGG pathways, and blue nodes indicate the genes that participated.

To explore the biological functions of Lut against ALI/ARDS, 84 genes were analyzed by GO analysis. The top 20 significantly enriched terms in GO and KEGG were selected, according to the analysis of GO term (MF) and KEGG pathway with pv < 0.05 and qv < 0.05. MF enrichment analysis was involved mainly with protein kinase and receptor ligand binding activity, which may be closely related to the pathological process of ALI/ARDS (Figure 7A).

The results of the KEGG pathways were related mainly to “PI3K/Akt”, “multiplevirus infections”, “JAK/STAT”, and “HIF-1” (Figure 7B). The critical pathway target visualization network is shown in Figure 7C; the corresponding targets of Lut for treatment of ALI were closely related to the inflammatory reaction, in which the targets were focused mainly on the JAK/STAT pathway.

### 2.8. Binding Activities of Lut to the JAK/STAT Pathway

**Figure 8:**
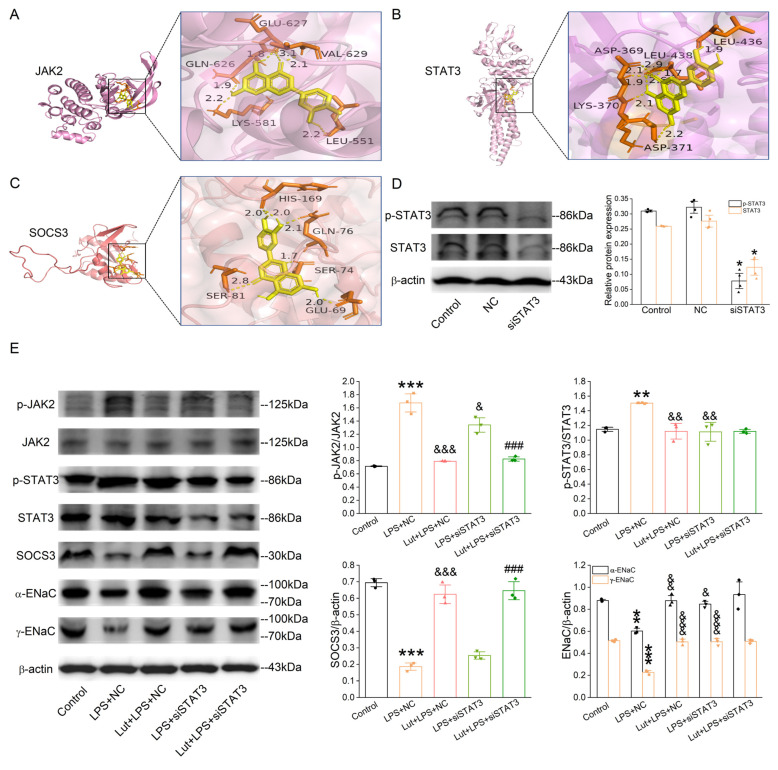
Lut suppressed the activation of JAK/STAT signaling pathway in 3D organoid culture. (**A**–**C**) Docking diagram of Lut with genes (JAK2, STAT3, and SOCS3) related to core pathway in KEGG of ALI. (**D**) Representative Western blot and corresponding graphical representation for p-STAT3/STAT3 of Control, NC (negative control), and siSTAT3 group in primary AT2 cells. * *p* < 0.05, compared with Control group, *n* = 4. Mann–Whitney U test was used to analyze the difference of the means for significance. (**E**) Representative Western blot and corresponding graphical representation for p-JAK2/JAK2, p-STAT3/STAT3, α/γ-EnaC, and SOCS3 in primary AT2 cells. ** *p* < 0.01, *** *p* < 0.001, compared with Control group. ^&^ *p* < 0.01, ^&&^ *p* < 0.01, ^&&&^ *p* < 0.001, compared with LPS + NC group, ^###^ *p* < 0.001, compared with LPS + siSTAT3 group, *n* = 3–4. One-way ANOVA followed by Bonferroni’s test was used to analyze the difference of the means for significance.

Lut was selected as a molecular ligand to dock with the JAK2, STAT3, and SOCS3 (component in the JAK/STAT pathway), respectively. As shown in Figure 8A–C, the affinities between the molecular ligand compound and the receptor proteins were all less than −5.0 kcal/mol, indicating that Lut possessed good binding activity with JAK2, STAT3, and SOCS3 proteins.

### 2.9. Validation of the Pathways and Targets

In order to verify the dependability of pathways and protein targets predicted by the network pharmacology and molecular docking simulations, we selected the key targets involved in the JAK/STAT pathway, including JAK2, STAT3, and SOCS3, for verification using Western blot assay. We knocked down STAT3 first in the primary AT2 cells, and the efficiency is shown in Figure 8D (*p* < 0.05 vs. Control group). LPS increased phospho-specific forms of JAK2 and STAT3 (Figure 8E, *p* < 0.001~0.01 vs. Control group), which were decreased after the Lut treatment (*p* < 0.001~0.01 vs. LPS + NC group), and the former was not affected by the knockdown of STAT3. Meanwhile, the LPS-reduced SOCS3 expression (*p* < 0.001 vs. Control group), a negative feedback regulator of JAK/STAT pathway, was inhibited by Lut with or without STAT3 knockdown (*p* < 0.001 vs. LPS + NC group or LPS + siSTAT3 group). As shown in Figure 8E, compared with that in Control, the α/γ-ENaC expression level in the LPS-treated AT2 cells was dramatically suppressed (*p* < 0.01~0.001 vs. Control group) and remarkably elevated by either the treatment of Lut or the lack of STAT3 (*p* < 0.001~0.05 vs. LPS + NC group). Intriguingly, the Lut-enhanced α/γ-ENaC expression was abolished after the co-administration of the STAT3 knockdown.

## 3. Discussion

Numerous lines of evidence indicate that exposure to LPS stimulation can elicit an inflammatory response and other pulmonary lesions, ultimately leading to the development of respiratory disorders. Serious pulmonary infection involved with LPS is a high risk factor for ALI/ARDS, which can cause inflammatory cells to recruit into the lung, an increase in capillary permeability, and alveolar edema [24,25,26]. The morphological changes caused by LPS were significant inflammatory cell infiltration, alleviation by hemorrhage, and thickness of the alveolar wall after the Lut treatment. In addition, the pulmonary W/D ratio, which illustrates the amount of exudate in the lungs, was significantly reduced, proving that Lut had a significant anti-edema effect on ALI. Our results also showed that Lut reduced the protein content and inflammatory cytokines in BALF after lung injury, suggesting that Lut alleviated the occurrence of LPS-induced protein-rich alveolar edema and protected the alveolar–capillary barrier.

Na^+^ enters AT2 cells mainly through apical ENaC, which is then pumped out by basolateral Na^+^ and K^+^-ATPase, accompanied by reabsorption of osmotic fluid [27,28,29]. The main determinant of alveolar fluid clearance is ENaC expressed on the apical side of AT2 cells [30,31]. In this study, primary AT2 cells of mice were isolated, and the results showed that Lut could reverse the LPS-induced decrease in ENaC expression at both the protein and transcription levels, suggesting that Lut could improve transepithelial sodium transport by upregulating ENaC expression.

However, the inability to maintain biological characteristics of primary AT2 cells during culture has been an important obstacle in the research of pulmonary biology. Primary AT2 cells can hardly be passaged by traditional culture methods, and they lose their characteristic cuboid-like appearance with the culture time. Meanwhile, the production of surface active substances decreases, and lamellar bodies are lost [32]. Compared with traditional 2D cell culture, the 3D alveolar epithelial organoids contain a variety of cell types and exhibit closer cellular interactions with the stroma, resulting in functional micro-organs that better simulate the physiological and pathological processes of organ tissues. We, thus, selected AT2 cells and MLg cells of mice to co-culture in matrix glue to construct the lung alveolar model using 3D alveolar epithelial organoids [11]. During the culture, we added a TGF-β inhibitor to prevent excessive proliferation of MLg cells, and we promoted the AT2 cell differentiation by inhibiting the TGF-β receptor signaling. The 3D alveolar epithelial organoids were certified through confocal images by co-expression of AT1 and AT2 cell markers (PDPN/SP-C), as well as alveolar epithelial structure by H&E staining. In addition, immunofluorescence and real-time PCR assay were performed on this model, which showed that Lut could reverse LPS-reduced ENaC expression at both the protein and transcription levels.

**Figure 9:**
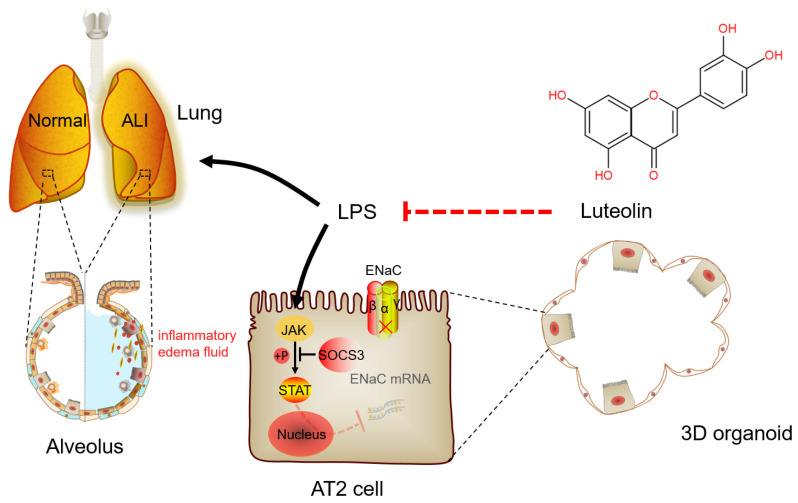
A schematic diagram for Lut to enhance the expression of ENaC in LPS-induced ALI. Lut can interdict the effect of LPS by increasing the expression of ENaC mRNA and protein via JAK/STAT pathway, as well as inhibiting the secretion of inflammatory cytokines in LPS-induced ALI. ALI, acute lung injury; AT2, alveolar epithelial type 2; ENaC, epithelial sodium channel; LPS, lipopolysaccharide.

LPS can activate the natural immune response and secrete a large number of inflammatory cytokines, such as TNF-α, IL-6, and IL-1β, the contents of which in BALF directly reflect the degree of inflammatory response in the alveolar cavity [33,34]. In addition, current research has suggested that epithelial cells are also involved in regulating inflammation and lung defense, in which AT2 cells serve as one of the main sources of neutrophil chemokines [35]. Our results showed that Lut had a significant anti-inflammatory effect on the LPS-induced mouse ALI model. Meanwhile, Lut reversed the LPS-reduced mRNA expression of IL-1β, IL-6, and TNF-α in primary mouse AT2 cells. The data proved that Lut could alleviate ALI through enhancing ENaC expression in both the primary AT2 cells and 3D alveolar epithelial organoid model, which lays a foundation for the mechanism of Lut relieving inflammation-related pulmonary edema and provides a potential therapeutic strategy for treating ALI (Figure 9).

The network pharmacology was applied to explore the inter-relationship between pharmaceuticals and diseases for various purposes, such as finding new drugs, elucidating pathogenesis, and discovering new targets [36,37,38]. We used the drug–disease network for topological analysis and applied the corresponding molecular docking method to improve the dependability of the target prediction results. Interactions between Lut and possible protein targets were predicted by combining messages from free databases relating ALI/ARDS, together with revealing the signaling networks in which the Lut targets participated. Hydrogen bonding seemed to be the main interaction form, according to the molecular docking analysis. Pathway relationship uncovered that the Lut-regulated signaling pathways contained mainly PI3K/Akt, multiple virus infections and JAK/STAT in ALI/ARDS. TP53, IL6, AKT1, SRC, JUN, EGFR, VEGFA, CASP3, ALB, JAK2, MAPK1, CCND1, RELA, and MMP9 were key genes with high degree values > 20. Through analyzing the Hub genes and the predominant KEGG pathways, the possible mechanisms of Lut in handling ALI/ARDS might be due to the JAK/STAT signaling pathway, which could modulate oxidative stress, cell apoptosis, and inflammation in an ALI model [39]. In our study, the treatment of Lut enhanced the SOCS3 expression and inhibited the phosphorylation level of JAK2 and STAT3 induced by LPS in the primary mouse AT2 cells. The knockdown of STAT3 did not affect the above effects of Lut on JAK2 phosphorylation or SOCS3 expression, indicating that they were upstream molecules for STAT3. Furthermore, siSTAT3 could abrogate the Lut enhancement of ENaC protein expression, implying a downstream mechanism involved with the STAT3 signal. Overall, Lut could attenuate inflammation-related ALI by enhancing transepithelial sodium transport, at least partially, via the JAK/STAT pathway.

## 4. Materials and Methods

### 4.1. Reagents

Lut (CAS: 491-70-3, purity 98%), LPS (purity 98%), and 4ʹ, 6-diamidino-2-phenylindole (DAPI) were purchased from Solarbio Science & Technology Co., Ltd. (Beijing, China). BCA protein detection and H&E kits were purchased from Beyotime Biological Co. (Shanghai, China). Dispase II, DNase I, and Matrigel were provided by Sigma-Aldrich (St. Louis, MO, USA). Low-melting-point agarose and primers were obtained from Sangon Biotech Co., Ltd. (Shanghai, China). Biotinylated antibodies CD16/32 and CD45 were provided by Miltenyi Biotec (Shanghai, China), and the information about specific antibodies are listed in Table 1. ELISA and CCK-8 kits were purchased from Neobioscience Technology Company (Shenzhen, China) and Biosharp Biological Technology Co., Ltd. (Guangzhou, China), respectively.

### 4.2. Animals

The Laboratory Animal Center of China Medical University provided the mice, with the certificate number: SYXK (Liao) 2018-0008. Animal experiments were conducted under the guidelines and regulations of Animal Care and Use Ethics Committee. Animals were anesthetized with diazepam (17.5 mg/kg), followed by ketamine (450 mg/kg).

### 4.3. Primary Mouse AT2 Cell Culture and Cell Viability Analysis

The primary AT2 cells were isolated from mice and cultured as described earlier [40]. Then, the separated primary cells were modulated to 2.5 × 10^6^ cells/mL in DMEM/F12 medium with FBS (10%, Gibco, New York, NY, USA), penicillin (100 IU), and streptomycin (100 μg/mL). The medium was replaced first after 72 h and then every other day thereafter.

The optimal concentration of Lut was detected by CCK8 assay [41]. The primary AT2 cells were incubated with Lut (0, 2.5, 5, 10, 20, and 40 μM) with or without LPS (10 μg/mL) for 24 h. After treatment, the viability of cells was measured by a CCK-8 kit (Biosharp, Guangzhou, China) according to the manufacturer’s protocol. In the following cellular experiments, the primary AT2 cells were treated with Lut (10 μM, 24 h) and/or LPS (10 μg/mL, 12 h), respectively.

### 4.4. Establishment of the ALI Mouse Model

The healthy BALB/c male mice with body weight of 20–25 g were randomly divided into the following 4 groups: Control, LPS, Lut, and Lut + LPS. ALI animal model was established by intraperitoneal injection of LPS (5 mg/kg, Solarbio, Beijing, China) [21]. The mice in Lut and Lut + LPS groups were intraperitoneally injected with Lut (70 μmol/kg, Solarbio, Beijing, China) twice, 12 h before and after LPS injection [42].

### 4.5. Preparation for 3D Organoid Culture

Adult mouse lungs were used for the isolation of AT2 cells [11]. In brief, the lung was perfused with 4 °C PBS. After trachea intubation, dispase II (3 mg/mL, Corning, New York, NY, USA) was injected into the lung, followed by 1% low-melting-point agarose (42–45 °C, Sangon Biotech, Shanghai, China). Then, the lung was incubated in dispase II for 45 min at 37 °C and gently torn in DMEM/F12 + DNase I (0.01%, Sigma, St. Louis, MO, USA). The cell suspension was passed through serial filters (Solarbio, Beijing, China) and incubated with biotinylated antibodies: CD16/32 and CD45 (Miltenyi Biotec, Shanghai, China) for 10 min. After being incubated with 10 µL Streptavidin MicroBeads (4 °C, Miltenyi Biotec, Shanghai, China) for 15 min in dark, the cells were transferred onto plates pre-coated with 1 mg/mL IgG for 2 h, and the unattached cells were collected. The cell purity was detected by flow cytometry with corresponding antibodies.

### 4.6. Identification of 3D Alveolar Epithelial Organoid

Mouse AT2 cells (6 × 10^3^) and MLg2908 cells (2 × 10^5^, American Type Culture Collection) were suspended in a 1:1 mixture of growth-factor-reduced Matrigel (Corning, New York, NY, USA) on polyester membrane cell culture transwells (0.4 μm pore size, 0.33 cm^2^ area, Corning, New York, NY, USA) and incubated for 30 min to allow the matrix to solidify.

After fixation, permeabilization, and blocking, the 3D organic cultures were incubated with SP-C and PDPN antibody overnight at 4 °C, respectively. The information about the antibodies are listed in Table 1. The nucleus was stained by DAPI. Finally, the sections were imaged by confocal microscopy.

### 4.7. Determination of Lung Wet/Dry Weight Ratio

The degree of pneumonedema was analyzed by lung W/D ratio to assess the severity of ALI. The lung W/D ratio was acquired by dividing the wet weight by the lung weight after 48 h of oven drying at 60 °C.

### 4.8. Bronchoalveolar Lavage Fluid Analysis

We collected the BALF to evaluate the extent of lung inflammation [43,44,45]. Alteration of the barrier between alveolar and capillary was evaluated depending on the BALF protein content via BCA kit (Beyotime Biotechnology, Shanghai, China), and the inflammatory cytokines were tested by ELISA assay (Neobioscience, Shenzhen, China).

### 4.9. Histological Studies

Freshly harvested lung tissues and 3D organoid cultures were fixed, dehydrated, and embedded in 4% paraformaldehyde, 30% sucrose, and OCT, respectively. To semi-quantify the histopathologic changes in H&E (Beyotime Biotechnology, Shanghai, China) staining, the Szapiel score was used to quantify alveolitis and determine the severity of lung injury through the score of 0 (no injury) to 3 (maximum injury) [46].

### 4.10. Real-Time PCR

The total RNA (1 μg) in samples was extracted and acquired by TRIzol (Invitrogen, Carlsbad, CA, USA) and used as the template for reverse transcription in triplicate. Real-time PCR was processed with the primers shown in Table 2. Reaction was processed with 95 °C (30 s), followed by 50 cycles of 95 °C (5 s), 52 °C (30 s), and 72 °C (60 s). The relative expression level of mRNA was obtained by 2^−Δ(ΔCT)^ comparative method.

### 4.11. Immunofluorescence Assay

The AT2 cells were incubated with primary α-ENaC and γ-ENaC antibodies, respectively, and then with secondary antibody (Table 1). After the nuclei were stained by DAPI, the sections were observed by a fluorescence microscope, and target proteins were quantified through Image J software.

### 4.12. Target Prediction

The potential targets of Lut were acquired from Traditional Chinese Medicine Systems Pharmacology (TCMSP, https://tcmsp-e.com/tcmsp.php), SwissTargetPredictive (http://www.swisstargetprediction.ch), and PharmMapper (http://www.lilab-ecust.cn/pharmmapper). By converting protein targets into gene targets by the database of UniProt (http://www.uniprot.org) and removing duplicate genes, we obtained 412 related genes.

The disease targets of ALI/ARDS were obtained from databases including DrugBank (https://go.drugbank.com), GeneCards (https://www.genecards.org, accessed on 3 November 2022), PharmGKb (https://www.pharmgkb.org), and Online Mendelian Inheritance in Man (OMIM, https://omim.org). The 1980/1935 related genes in ALI/ARDS were obtained by merging the 4 disease database targets and deleting the duplicates.

### 4.13. Constructing Protein–Protein Interaction Networks

The intersection between disease and drug targets was captured by Venny 2.1 (https://bioinfogp.cnb.csic.es/tools/venny), and the Venn diagram was drawn. There were 84 common targets between Lut and ALI/ARDS.

By using the limitation to “Homo sapiens” and confidence score ≥ 0.7, the 84 overlapped target PPI networks were obtained by the database of String (https://string-db.org). The PPI network containing the degree values of nodes was generated by Cytoscape v3.7.2. In Cytohubba plugin of CytoScape, PPI network Hub genes for Lut against ALI/ARDS were calculated by MCC algorithm, and 10 core targets were acquired to construct the related protein target networks. The major dense regions were selected from the network of 84 overlapped genes using Cytoscape plugin Molecular Complex Detection, in which seed genes were present in clusters with the largest MCODE Score. The following settings were used: Degree Cut-off = 2, Node Score Cut-off = 0.2, K-Core = 2, and Max Depth = 100.

### 4.14. Pathway Enrichment Analysis

Annotation, Visualization, Integrated Discovery (https://david.ncifcrf.gov), and Kyoto Encyclopedia of Genes and Genomes (KEGG, www.kegg.jp/kegg) were used for pathway enrichment analysis. *p* < 0.05 in KEGG enrichments was considered significant.

### 4.15. Molecular Docking Analysis

The docking ligand of Lut 3D structure in PDBQT format was obtained from the PubChem database and Chem3D software. PyMol (https://pymol.org/2) was used to delete water molecules and add hydrogen bonds of protein conformation, which was acquired from the PDB (https://www.rcsb.org). AutoDock Tools V1.5.6 (http://autodock.scripps.edu) software was used to detect the roots, set the rotatable key, calculate the Gasteiger charge, and identify the binding pockets. Then, AutoDockVinaV1.1.2 was used for molecular docking, and the results were visualized using PyMol software.

### 4.16. Western Blot Assay

Proteins were separated and transferred to PVDF membranes (Invitrogen, Waltham, MA, USA), which were then incubated with primary and secondary antibodies, respectively. The information about the antibodies is also listed in Table 1. Finally, the protein bands were visualized by ECL kit and quantitatively analyzed by the Image J.

### 4.17. Statistical Analysis

We applied Origin 2021 software to process the data as mean ± SE and assessed the power of sample size firstly to meet *p* < 0.05. When the data passed the Levene’s test for normality and Shapiro–Wilk’s test for heteroscedasticity, one-way analysis of variance (ANOVA) and Bonferroni’s test was used for comparison among multiple groups. If the data did not pass the normality and heteroscedasticity tests, the non-parametric *t*-test (Mann–Whitney U test) was applied.

## Figures and Tables

**Table 1:** The information about the antibodies.

Antibody	Manufacture	WB	IF	FCM
SP-C	Bioss (Beijing, China)		1:100	1:100
PDPN	Santa Cruz (Dallas, TX, USA)		1:100	
p-STAT3	Affinity (Cincinnati, OH, USA)	1:1000		
p-JAK2	Affinity (Cincinnati, OH, USA)	1:1000		
STAT3	Affinity (Cincinnati, OH, USA)	1:1000		
JAK2	Affinity (Cincinnati, OH, USA)	1:1000		
SOCS3	Affinity (Cincinnati, OH, USA)	1:1000		
α-ENaC	Santa Cruz (Dallas, TX, USA)	1:2000	1:200	
γ-ENaC	Santa Cruz (Dallas, TX, USA)	1:2000	1:200	
β-actin	Santa Cruz (Dallas, TX, USA)	1:1000		
goat anti-mouse	ZSGB-BIO (Beijing, China)	1:5000		
goat anti-rabbit	ZSGB-BIO (Beijing, China)	1:5000		
FITC goat anti-rabbit	ZSGB-BIO (Beijing, China)		1:100	1:200
TRITC goat anti-mouse	ZSGB-BIO (Beijing, China)		1:100	

WB: Western blot, IF: immunofluorescence, FCM: flow cytometry.

**Table 2:** The information about the primers.

Primer Name	Forward (5′–3′)	Reverse (5′–3′)
α-ENaC	AAC AAA TCG GACTGC TTC TAC	AGC CAC CAT CAT CCA TAA A
β-ENaC	GGG ACC AAA GCA CCA AT	CAG ACG CAG GGA GTC ATAG
γ-ENaC	GCACCG TTC GCC ACC TTC TA	AGG TCA CCA GCA GCT CCT CA
IL-1β	AGA AGC TGT GGC AGC TAC CTG	GGA AAA GAA GGT GCT CAT GTC C
IL-6	GCT ACC AAA CTG GAT ATA ATC AGG A	CCA GGT AGC TAT GGT ACT CCA GAA
TNF-α	TCT TCT CAT TCC TGC TTG TGG	GGT CTG GGG CCA TAG AAC TGA
GAPDH	AGA AGG CTG GGG CTC ATT TG	AGG GGC CAT CCA CAG TCT TC

## Data Availability

All data/code generated or analyzed during this study are available from the corresponding author on reasonable request.
